# Author Correction: Dynamic forecasting of severe acute graft-versus- host disease after transplantation

**DOI:** 10.1038/s43588-022-00256-7

**Published:** 2022-05-03

**Authors:** Xueou Liu, Yigeng Cao, Ye Guo, Xiaowen Gong, Yahui Feng, Yao Wang, Mingyang Wang, Mengxuan Cui, Wenwen Guo, Luyang Zhang, Ningning Zhao, Xiaoqiang Song, Xuetong Zheng, Xia Chen, Qiujin Shen, Song Zhang, Zhen Song, Linfeng Li, Sizhou Feng, Mingzhe Han, Xiaofan Zhu, Erlie Jiang, Junren Chen

**Affiliations:** 1 State Key Laboratory of Experimental Hematology, National Clinical Research Center for Blood Diseases, Institute of Hematology & Blood Diseases Hospital, Chinese Academy of Medical Sciences & Peking Union Medical College, Tianjin, China; 2 Yidu Cloud Technology Inc., Beijing, China

**Keywords:** Computational science, Computational models, Graft-versus-host disease

Correction to: *Nature Computational Science* 10.1038/s43588-022-00213-4, published online 28 March 2022.

At the time this article was originally published, the authors’ application to deposit a desensitized aGOAT dataset with the PRC National Genomics Data Center was still under review. After publication of this work, the Chinese government has approved the archiving of the aGOAT dataset at the PRC National Genomics Data Center (NGDC) in April, 2022 (ref. no. 2022BAT1224). Accordingly, the authors have made the dataset publicly accessible at the NGDC website (https://ngdc.cncb.ac.cn/omix/release/OMIX001095/). The Data availability statement has been amended in the HTML and PDF versions of the article.

