# Supplementary material for: Dynamic forecasting of severe acute graft-versus-host disease after transplantation
Source: Nat Comput Sci. 2022 Mar 28;2(3):153–9. doi: 10.1038/s43588-022-00213-4 (PMC10766514; doi:10.1038/s43588-022-00213-4)
Supplement: Supplementary file 1 — Supplementary Figs. 1–3 [file 43588_2022_213_MOESM1_ESM.pdf]

nature portfolio

https://doi.org/10.1038/s43588-022-00213-4

Supplementary information

Dynamic forecasting of severe acute graft-versus-host disease after transplantation

In the format provided by the authors and unedited

Supplementary Information

Dynamic forecasting of severe acute graft-versus-host disease after transplantation

Xueou Liu[1,3], Yigeng Cao[1,3], Ye Guo[1,3], Xiaowen Gong[1,3], Yahui Feng[1,3], Yao Wang[2,3], Mingyang Wang[1], Mengxuan Cui[2], Wenwen Guo[1], Luyang Zhang[1], Ningning Zhao[1], Xiaoqiang Song[1], Xuetong Zheng[1], Xia Chen[1], Qiujin Shen[1], Song Zhang[1], Zhen Song[1], Linfeng Li[2], Sizhou Feng[1], Mingzhe Han[1], Xiaofan Zhu[1*], Erlie Jiang[1*], Junren Chen[1*]

[1] State Key Laboratory of Experimental Hematology, National Clinical Research Center for Blood Diseases, Institute of Hematology & Blood Diseases Hospital, Chinese Academy of Medical Sciences & Peking Union Medical College, Tianjin, China.

[2] Yidu Cloud Technology Inc., Beijing, China.

[3] These authors contributed equally to this work.

[*] Corresponding authors.

This PDF file includes:

Supplementary Figs. 1 to 3

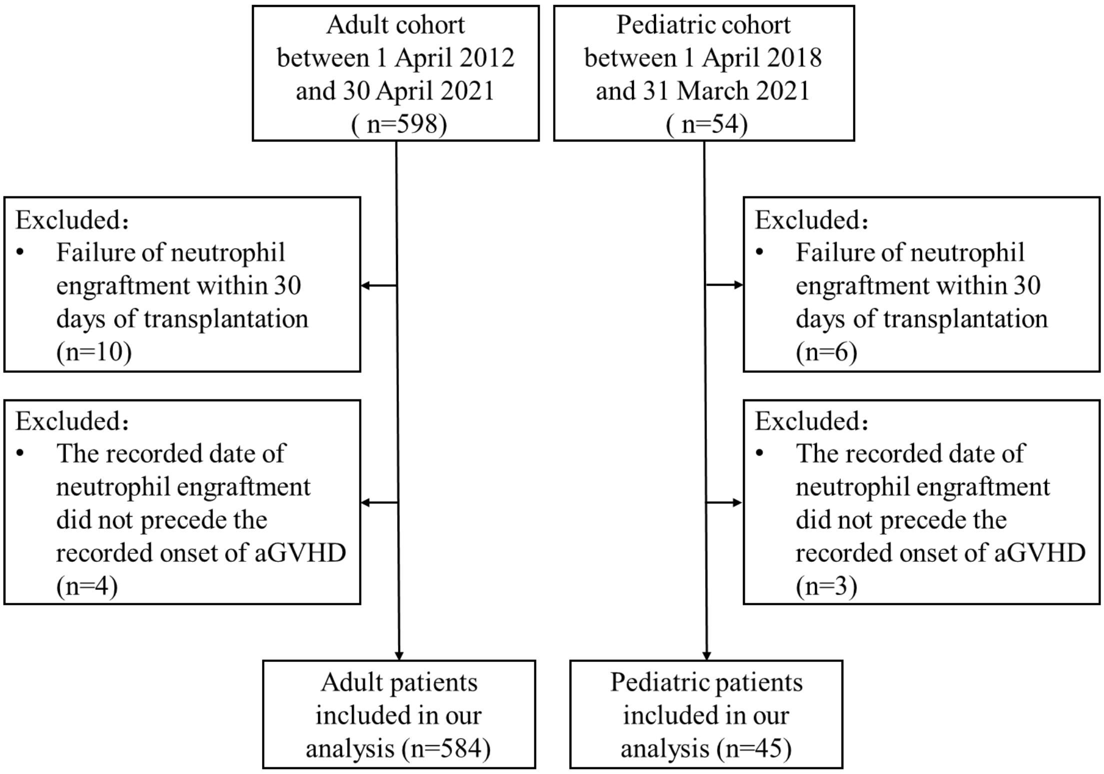

Supplementary Fig. 1. Patient screening flow chart.

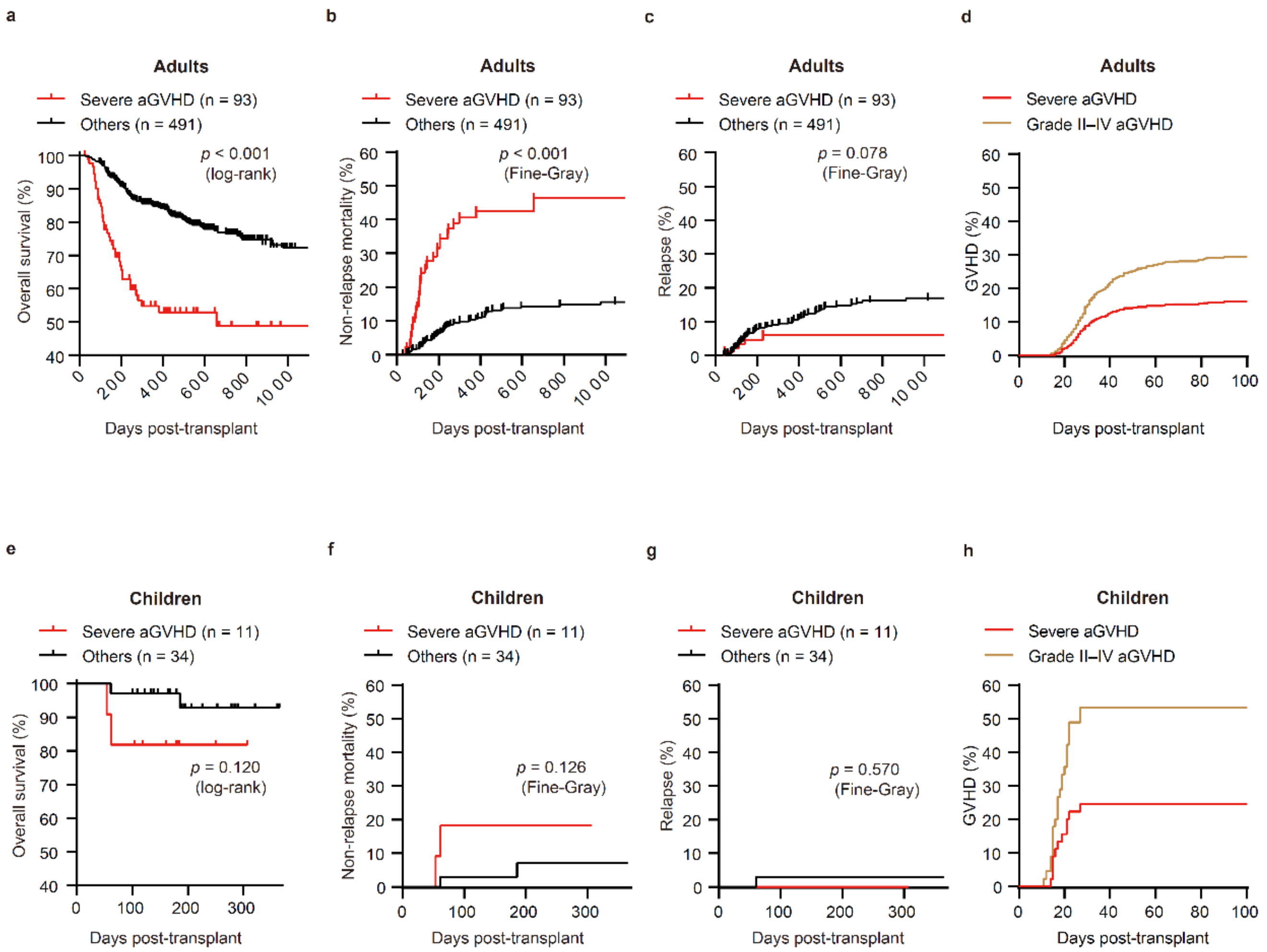

Supplementary Fig. 2. Overall survival and cumulative incidence curves for NRM, relapse, severe aGVHD, and grade II–IV aGVHD in the adult (**a–d**) and the pediatric (**e–h**) cohorts. Overall survival curves (**a,e**) were estimated by the Kaplan-Meier method. When calculating cumulative incidence curves for NRM (**b,f**) and relapse (**c,g**), NRM and relapse were treated as mutually exclusive competing events for each other. When calculating cumulative incidence curves for aGVHD (**d,h**), all-cause death was treated as a competing event that precluded aGVHD. (Red, severe aGVHD cases; black, cases that did not develop severe aGVHD; gold, grade II–IV aGVHD cases.)

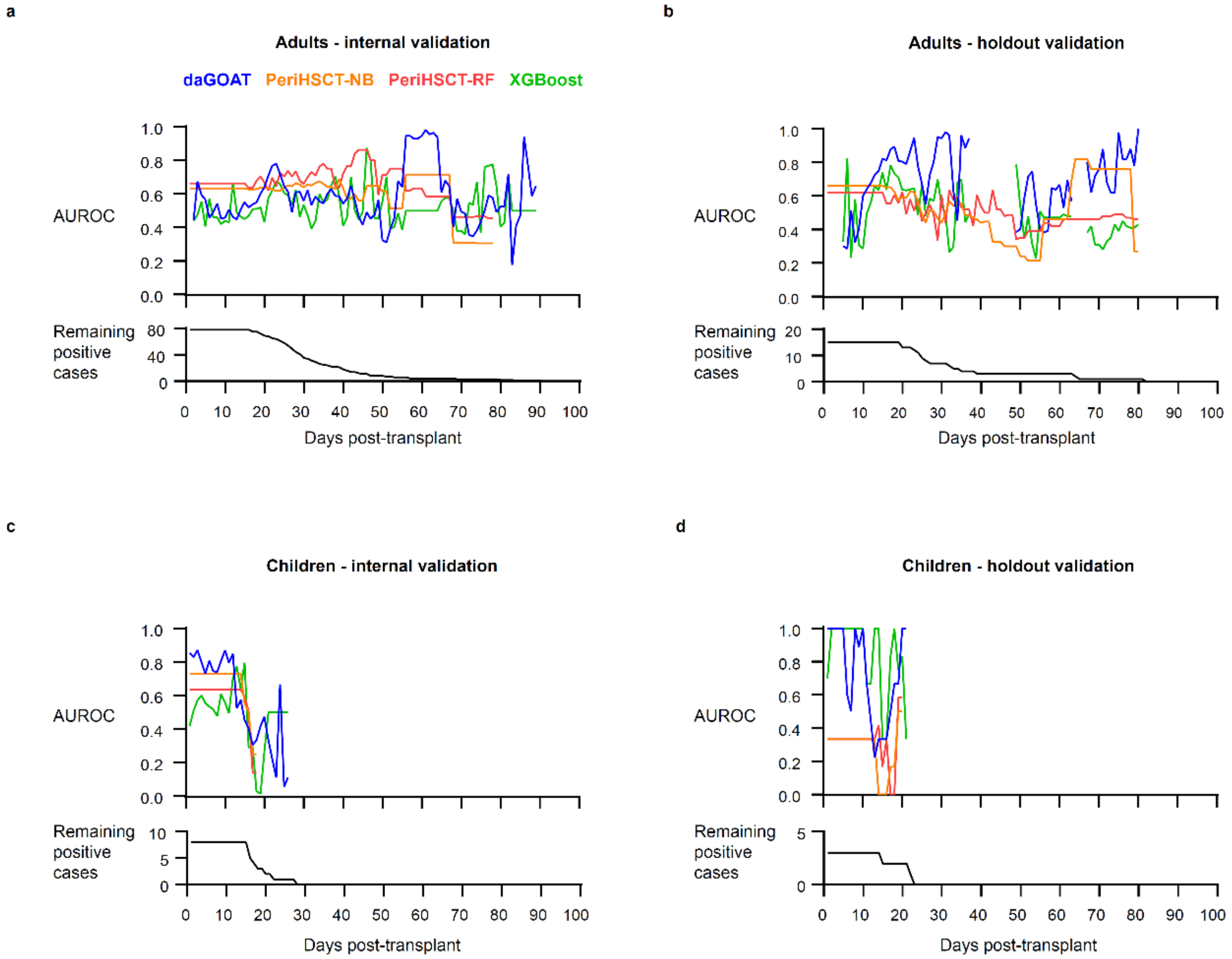

Supplementary Fig. 3. Temporal profiles for AUROC in internal and holdout validations for the adult (**a,b**) and pediatric (**c,d**) cohorts. **a,c,** Internal validations. **b,d,** Holdout validations. (Blue, daGOAT; orange, PeriHSCT-NB; red, PeriHSCT-RF; green, XGBoost.) The bottom panels show how the number of remaining positive cases (severe aGVHD cases that had not had onset) decreased over time in the patient cohorts.
