# Supplementary material for: Dynamic forecasting of severe acute graft-versus-host disease after transplantation
Source: Nat Comput Sci. 2022 Mar 28;2(3):153–9. doi: 10.1038/s43588-022-00213-4 (PMC10766514; doi:10.1038/s43588-022-00213-4)
Supplement: Supplementary file 2 — Reporting Summary [file 43588_2022_213_MOESM2_ESM.pdf]

nature research

Corresponding author(s): Junren Chen, Erlie Jiang, Xiaofan Zhu

Last updated by author(s): Feb 12, 2022

Reporting Summary

Nature Research wishes to improve the reproducibility of the work that we publish. This form provides structure for consistency and transparency in reporting. For further information on Nature Research policies, see our Editorial Policies and the Editorial Policy Checklist.

Statistics

For all statistical analyses, confirm that the following items are present in the figure legend, table legend, main text, or Methods section.

n/a	Confirmed	
☐	☒	The exact sample size (n) for each experimental group/condition, given as a discrete number and unit of measurement
☐	☒	A statement on whether measurements were taken from distinct samples or whether the same sample was measured repeatedly
☐	☒	The statistical test(s) used AND whether they are one- or two-sided<br>*Only common tests should be described solely by name; describe more complex techniques in the Methods section.*
☐	☒	A description of all covariates tested
☐	☒	A description of any assumptions or corrections, such as tests of normality and adjustment for multiple comparisons
☐	☒	A full description of the statistical parameters including central tendency (e.g. means) or other basic estimates (e.g. regression coefficient) AND variation (e.g. standard deviation) or associated estimates of uncertainty (e.g. confidence intervals)
☐	☒	For null hypothesis testing, the test statistic (e.g. F, t, r) with confidence intervals, effect sizes, degrees of freedom and P value noted<br>*Give P values as exact values whenever suitable.*
☒	☐	For Bayesian analysis, information on the choice of priors and Markov chain Monte Carlo settings
☒	☐	For hierarchical and complex designs, identification of the appropriate level for tests and full reporting of outcomes
☐	☒	Estimates of effect sizes (e.g. Cohen's d, Pearson's r), indicating how they were calculated

Our web collection on statistics for biologists contains articles on many of the points above.

Software and code

Policy information about availability of computer code

Data collection	All data used in this study were retrieved from the electronic health record system at the Institute of Hematology, Chinese Academy of Medical Sciences (IHCAMS). Custom scripts used for accessing the hospital's internal database and for curating the retrieved data have to remain confidential.
Data analysis	R code used in this study is available in a public GitHub repository at https://github.com/chenjunren-ihcams/daGOAT (DOI: 10.5281/zenodo.6041841). Utilized R libraries and versions: readxl (ver. 1.3.1), dplyr (ver. 1.0.8), e1071 (ver. 1.7-9), randomForest (ver. 4.7-1), xgboost (ver. 1.5.0.2), pROC (ver. 1.18.0), and PRROC (ver. 1.3.1).

For manuscripts utilizing custom algorithms or software that are central to the research but not yet described in published literature, software must be made available to editors and reviewers. We strongly encourage code deposition in a community repository (e.g. GitHub). See the Nature Research guidelines for submitting code & software for further information.

Data

Policy information about availability of data

All manuscripts must include a data availability statement. This statement should provide the following information, where applicable:

- Accession codes, unique identifiers, or web links for publicly available datasets
- A list of figures that have associated raw data
- A description of any restrictions on data availability

While there was no genetic polymorphism, gene expression, or protein sequence data involved in this study, sharing of substantial clinical data generated from China's human genetic resources needs to abide by the Regulations of the People's Republic of China (PRC) on the Administration of Human Genetic Resources. A 'minimum dataset' for severe aGVHD that would be necessary to verify the research in this article would include all dynamic features (from day 1 through day 100 post-HSCT) listed in Supplementary Table 2, all peri-transplantation features listed in Supplementary Table 4, presence/absence of severe aGVHD within 100 days,

and onset dates of severe aGVHD. On 21 February 2022, the PRC Human Genetic Resources Administration Office approved the compilation of a desensitized version of the 'minimum aGOAT dataset' for facilitating international research collaborations (Reference No. CJ0272 (2022)). At the time of the publication of the manuscript, the authors' application to deposit this desensitized dataset at the PRC National Genomics Data Center (NGDC) database is still under review. To inquire the latest status of public accessibility to the desensitized 'minimum aGOAT dataset' at the NGDC database, please contact Junren Chen (chenjunren@ihcams.ac.cn). A mock-up dataset that can be used for demo runs of the daGOAT algorithm is available in a public Zenodo repository at https://zenodo.org/record/6050675#.Ygcg1N_P2Uk (DOI: 10.5281/zenodo.6050675). The waist-mounted smartphone inertial sensor dataset is available from the UCI Machine Learning Repository (https://archive.ics.uci.edu/ml/datasets/Smartphone-Based+Recognition+of+Human+Activities+and+Postural+Transitions). Source data for Figs. 1 and 2 and Extended Data Figs. 1–5 are provided with this paper.

Field-specific reporting

Please select the one below that is the best fit for your research. If you are not sure, read the appropriate sections before making your selection.

☒ Life sciences ☐ Behavioural & social sciences ☐ Ecological, evolutionary & environmental sciences

For a reference copy of the document with all sections, see nature.com/documents/nr-reporting-summary-flat.pdf

Life sciences study design

All studies must disclose on these points even when the disclosure is negative.

Sample size	Post-transplant multidimensional time-series clinical data of 584 adult patients (age >16) who received HLA-mismatched allo-HSCT with stem cells sourced from peripheral blood, bone marrow, or both between 1 April 2012 and 30 April 2021 and 45 pediatric patients (age ≤16) who received HLA- mismatched allo-HSCT with stem cells sourced from peripheral blood, bone marrow, or both between 1 April 2018 and 31 March 2021 at the IHCAMS were able to be electronically retrieved and curated.
Data exclusions	16 cases (10 adults and 6 children) were eliminated due to failure of neutrophil engraftment within 30 days of transplantation. Additional 7 cases (4 adults and 3 children) were eliminated, because the recorded date of neutrophil engraftment (defined as 'the date of the first of three consecutive measurements spanning ≥3 days of achieving a sustained peripheral blood neutrophil count of >500×10^6/L') did not precede the recorded onset of aGVHD.
Replication	The same modeling approach was independently applied to both the adult and pediatric cohorts, and in both cohorts both internal validation and holdout validation were independently performed. Our main finding – i.e., daGOAT outperformed the benchmarks – was replicated in all four scenarios (adult-internal validation, adult-holdout validation, children-internal validation, children-holdout validation).
Randomization	Not applicable. There was no experiment performed on any human subject. This study was a retrospective analysis on patients treated at the IHCAMS.
Blinding	Not applicable. There was no experiment performed on any human subject. This study was a retrospective analysis on patients treated at the IHCAMS.

Reporting for specific materials, systems and methods

We require information from authors about some types of materials, experimental systems and methods used in many studies. Here, indicate whether each material, system or method listed is relevant to your study. If you are not sure if a list item applies to your research, read the appropriate section before selecting a response.

Materials & experimental systems

n/a	Involved in the study	
☒	☐	Antibodies
☒	☐	Eukaryotic cell lines
☒	☐	Palaeontology and archaeology
☒	☐	Animals and other organisms
☐	☒	Human research participants
☒	☐	Clinical data
☒	☐	Dual use research of concern

Methods

n/a	Involved in the study	
☒	☐	ChIP-seq
☒	☐	Flow cytometry
☒	☐	MRI-based neuroimaging

Human research participants

Policy information about studies involving human research participants

Population characteristics	The adult cohort contained 584 patients with the following characteristics: age 17-64 (median 35), male sex 58%, primarily myeloid neoplasms (AML (44%), ALL (22%), MDS (20%), aplastic anemia (9%)), and receiving primarily (97%) haploidentical hematopoietic stem cell transplantation.<br><br>The pediatric cohort contained 45 patients with the following characteristics: age 2-15 (median 9), male sex 69%, primarily

	bone marrow failure diseases (47%) and myeloid neoplasms (49%), and all (100%) receiving haploidentical hematopoietic stem cell transplantation.
Recruitment	629 consecutive cases whose data could be retrieved were included in this study. 23 cases were excluded from this study during QA/QC (see 'Data exclusions' on p. 2).
Ethics oversight	This retrospective study was approved by the IHCAMS Clinical Research Academic Committee on 11 January 2021 (IIT2021006) and by the IHCAMS Ethics Committee on 7 February 2021 (IIT2021006-EC-1). All the patients included in this study signed an informed consent form that permitted their biological samples or data to be utilized for research.

Note that full information on the approval of the study protocol must also be provided in the manuscript.
